# Blood–Brain Barrier Integrity Is Perturbed in a *Mecp2*-Null Mouse Model of Rett Syndrome

**DOI:** 10.3390/biom13040606

**Published:** 2023-03-28

**Authors:** Giuseppe Pepe, Salvatore Fioriniello, Federico Marracino, Luca Capocci, Vittorio Maglione, Maurizio D’Esposito, Alba Di Pardo, Floriana Della Ragione

**Affiliations:** 1 IRCCS Neuromed, 86077 Pozzilli, Italy; 2 Institute of Genetics and Biophysics ‘A. Buzzati-Traverso’, CNR, 80131 Naples, Italy

**Keywords:** Rett syndrome, blood–brain barrier, MeCP2, neurodevelopmental disorder, autism spectrum disorder

## Abstract

Rett syndrome (RTT, online MIM 312750) is a devastating neurodevelopmental disorder characterized by motor and cognitive disabilities. It is mainly caused by pathogenetic variants in the X-linked *MECP2* gene, encoding an epigenetic factor crucial for brain functioning. Despite intensive studies, the RTT pathogenetic mechanism remains to be fully elucidated. Impaired vascular function has been previously reported in RTT mouse models; however, whether an altered brain vascular homeostasis and the subsequent blood–brain barrier (BBB) breakdown occur in RTT and contribute to the disease-related cognitive impairment is still unknown. Interestingly, in symptomatic *Mecp2*-null (*Mecp2*^-/y^*, Mecp2*^tm1.1Bird^) mice, we found enhanced BBB permeability associated with an aberrant expression of the tight junction proteins *Ocln* and *Cldn*-5 in different brain areas, in terms of both transcript and protein levels. Additionally, *Mecp2*-null mice showed an altered expression of different genes encoding factors with a role in the BBB structure and function, such as *Cldn3*, *Cldn12*, *Mpdz*, *Jam2*, and *Aqp4*. With this study, we provide the first evidence of impaired BBB integrity in RTT and highlight a potential new molecular hallmark of the disease that might open new perspectives for the setting-up of novel therapeutic strategies.

## 1. Introduction

Rett syndrome (RTT, online MIM 312750) is a rare and devastating genetic neurodevelopmental disorder that affects almost exclusively females [1,2]. The disease is characterized by normal early growth during the first months of life, followed by a progressive slowing of development with subsequent intellectual disability, microcephaly, onset of autistic signs and seizures, and ultimately resulting in severe cognitive impairment and overall physical disabilities [3,4].

Mutations in the X-linked methyl-CpG binding protein 2 (*MECP2*) gene [5,6,7] have been reported to account for more than 95% of classical RTT cases [8]. *MECP2* encodes an epigenetic modulator of transcription [9,10,11] with a key role in heterochromatin architecture [12,13,14], which is expressed in a plethora of cell types and tissues, including endothelial cells and the circulatory system [15].

Besides the most common functional abnormalities described in RTT pathology, recently a significantly impaired vascular function has been highlighted in a mouse model of the disease [16,17]. Defective vascular homeostasis is increasingly recognized to strongly contribute to cognitive impairment in different pathological conditions [18,19,20]. However, whether such a dysfunction occurs in RTT and whether it has a role in the severe cognitive impairment observed in patients [21,22] and mouse models [23,24] remains to be elucidated.

The disrupted brain vasculature participates in the blood–brain barrier (BBB) breakdown, which has been described in several neurodegenerative disorders, such as Alzheimer’s disease (AD), Parkinson’s disease (PD), and Huntington’s disease (HD) [25,26,27,28,29,30,31], associated with cognitive decline. Similar dysfunction has been recently reported in the autism spectrum disorders (ASDs) [32,33], a group of neurodevelopmental diseases that includes RTT, and in other forms of neuropsychiatric diseases [34,35]. The BBB plays a crucial role in the central nervous system’s (CNS) defense. It is a highly dynamic and specialized structure that acts as a biochemical barrier to protect the brain from the inadequate access of circulating solutes and/or macromolecules that could negatively impact the neuronal activity and the overall health of the nervous system. Besides serving as a physical barrier, the BBB is critical for the homeostasis of metabolites and nutrients commonly used by the brain for its functioning. The maintenance of such homeostasis is crucial to also allow the excretion from the brain to the blood of metabolites that can be neurotoxic at high levels [36]. To this regard, any functional and/or structural alteration of the BBB is expected to have detrimental consequences on brain metabolism.

A perturbed permeability of BBB has been found to precede the onset of overt clinical signs in different neuropathological conditions, such as HD and AD [26,37], and it has been recently defined as an early biomarker of human cognitive dysfunction [38].

Our group has contributed to describe a functional impairment of BBB in a mouse model of HD and to demonstrate that the deregulation of tight junction (TJ) proteins, with the consequent alteration of the endothelial permeability of FITC-albumin, represents the underlying pathological mechanism [26,39].

In this study, we investigated whether there exists any link between BBB and RTT, for which no evidence of perturbed BBB homeostasis has ever been reported. In particular, we analyzed the BBB integrity in the *Mecp2*-null (*Mecp2*^-/y^*, Mecp2*^tm1.1Bird^) mouse model, one of the most widely used animal models of RTT, which recapitulates many features of the human pathology [40].

Interestingly, here, the evidence of BBB leakage and the abnormal expression of specific TJs (physiologically implicated to provide structural and functional support to the BBB) highlighted for the first time a yet unexplored interplay between BBB disruption and RTT pathology in *Mecp2*-null mice.

To the best of our knowledge, the evidence of major alterations of BBB permeability in RTT mouse models represents, absolutely, the first indication of a pathophysiological role of BBB in RTT and suggests that its disruption might contribute to the complex phenotypic scenario of the disease.

## 2. Materials and Methods

### 2.1. Animal Models

Heterozygous *B6.129P2(C)-Mecp2*^tm1.1Bird^/J female (*Mecp2*^+/-^) mutant mice, carrying the deletion of exon 3 and part of the exon 4 of the *Mecp2* gene, and wild-type (WT, C57BL⁄6) male mice were originally purchased from Jackson Laboratory (Bar Harbor, ME, USA) and then bred in the animal facility at IRCCS Neuromed. Heterozygous *Mecp2*^+/-^ female mice were crossed with WT male mice for the maintenance of the colony. Mice were housed individually in a temperature- and humidity-controlled environment under a 12-h light/dark cycle with ad libitum access to food and water.

Genotyping was performed by routine PCR techniques according to Jackson Laboratory protocols. All animal studies were performed in accordance with approved protocols by the IRCCS Neuromed Animal Care Review Board and by “Istituto Superiore di Sanità” (permit number: 988/2015-PR) and were conducted according to EU Directive 2010/63/EU for animal experiments. All the experimental procedures were performed on symptomatic (7–9 weeks) *Mecp2*^-/y^ mutant male mice [41] and age- and gender-matched WT littermates.

### 2.2. Evaluation of BBB Integrity and Analysis of FITC-Albumin/Laminin by Immunohistochemistry

The BBB integrity was evaluated by using FITC-albumin, which is a dye capable of crossing the blood–brain barrier only when it is not intact. Mice were treated with an intra-jugular injection of FITC-albumin (10 mg/mL in PBS at 10 mL/kg) (Sigma-Aldrich, St. Louis, MO, USA; Cat. N. A9771–100 MG) and were decapitated twelve minutes after infusion. The brain was immediately frozen in cool isopentane and was cut serially with a Cryostat (Leica Instruments, Wetzlar, Germany) in 10 µm thick sections. Brain sections were incubated with anti-laminin (1:1000) (Novus Biological, Centennial, CO, USA; Cat N. NB300–144) overnight at 4 °C and then with anti-mouse IgG secondary antibody (Vector-Laboratories, Newark CA, USA) for 1 h at room temperature. BBB integrity was evaluated as previously described [26,42]. Two mice per experimental group were used and four coronal sections for each animal were scanned and analyzed by ImageJ software.

### 2.3. Immunoblottings

Mice were sacrificed by cervical dislocation; then, the cortex, the striatum, the cerebellum, and the hippocampus were dissected out. Tissues were homogenized in a lysis buffer containing 20 mM Tris, pH 7.4, 1% Nonidet P-40, 1 mM EDTA, 20 mM NaF, and 2 mM Na3VO4 and a 1:1000 protease inhibitor mixture (Sigma-Aldrich) and sonicated with 2 × 10 s pulses. For claudin-5 and occludin, 20 µg of total protein lysate was immunoblotted with the following antibodies: anti-claudin-5 (1:1000) (Abcam, Cambridge, UK; Cat. N. ab15106) and anti-occludin (1:1000) (ThermoFisher Scientific, Waltham, MA, USA; Cat. N. 710192). For protein normalization, anti-actin (1: 5000) (Sigma-Aldrich; Cat. N. A5441) was used. Protein bands were detected by ECL Prime (GE Healthcare, Chicago, IL, USA) and quantitated with Quantity One Software (Bio-Rad Laboratories, Hercules, CA, USA).

### 2.4. RNA Extraction and Reverse-Transcription qPCR

Total RNA was extracted using an RNeasy kit (Qiagen, Hilden, Germany) according to the manufacturer’s instructions. The amount of 500 ng of total RNA was reverse-transcribed using Super Script III reverse transcriptase (ThermoFisher Scientific) and the resulting cDNA was amplified using Power SYBR green PCR master mix (Bio-Rad) following the manufacturer’s instructions. Quantitative PCR (qPCR) analysis was performed using the primers reported in Table 1.

### 2.5. Statistics

A two-tailed unpaired *t*-test and the Mann–Whitney test were used as indicated. All data were expressed as mean ± SD.

## 3. Results

### 3.1. Blood–Brain Barrier Permeability Is Altered in Symptomatic RTT Mecp2^-/y^ Mice

Several studies indicate that BBB is of fundamental importance for the maintenance of brain homeostasis and proper neuronal function [43,44]. The disruption of BBB is implicated in different brain disorders, including neurodegenerative disorders [25,26,27,28,29,30,31], psychiatric diseases [34,35], and ASDs [32,34].

**Figure 1:**
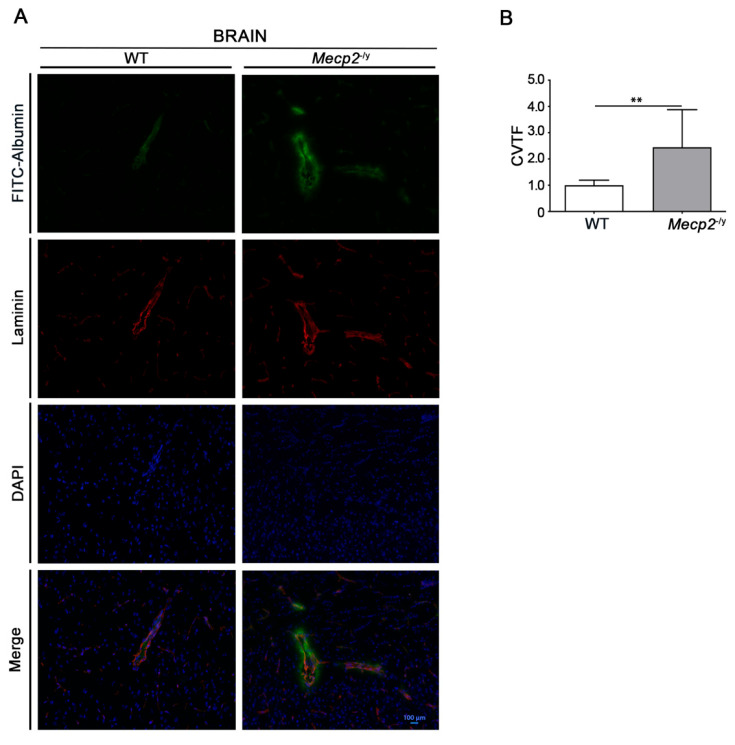
BBB disruption in *Mecp2*^-/y^ mouse model. Representative fluorescence micrographs (**A**) of brain cryosections from symptomatic *Mecp2*^-/y^ mice and age-matched WT littermates; semi-quantitative analysis (**B**) of FITC-albumin extravasation (bright green fluorescence) normalized on vessel marker laminin (red). Data are represented as mean ± SD from eight coronal sections from two different mice. **, *p*  <  0.01; (Mann–Whitney test). CTVF—corrected total vessel fluorescence.

In order to assess any sign of BBB leakage in RTT mice, we investigated tissue fluorescence for the tracer FITC-albumin and the vascular marker laminin in freshly frozen brain tissues from symptomatic (7–9 weeks old) *Mecp2*^-/y^ mice and age-matched WT littermates. Microscopy analysis revealed an increased permeability of the BBB, testified by a diffuse fluorescence along perivascular spaces and brain parenchyma in brain slices from RTT mice, whereas no visible sign of FITC-albumin extravasation was observed in WT mice (Figure 1A,B).

### 3.2. Expression of Claudin-5 and Occludin Proteins Is Decreased in Brain Tissues of Symptomatic Mecp2^-/y^ Mice

**Figure 2:**
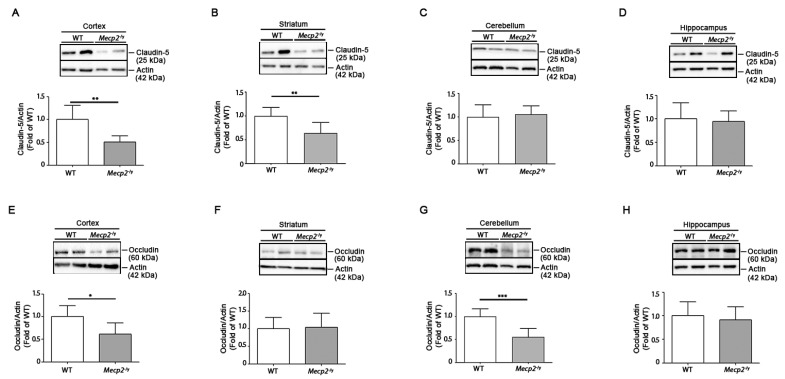
Expression of claudin-5 and occludin proteins is defective in brain tissues of *Mecp2*^-/y^ mice. Representative immunoblotting and densitometric analysis of claudin-5 and occludin in cortex (**A**,**E**), striatum (**B**,**F**), cerebellum (**C**,**G**), and hippocampus (**D**,**H**) of *Mecp2*^-/y^ mice and age-matched WT littermates. Actin was used for normalization. Values are represented as mean ± SD. N = 6/7 for each group of mice. *, *p* < 0.05; **, *p* < 0.01; ***, *p* < 0.001 (two-tailed unpaired *t*-test). Actin blots in (**C**,**G**) derived from the same gel. Actin blots in (**D**,**H**) derived from the same gel.

Alterations in the expression of TJ proteins, claudin-5 and occludin, are associated with an aberrant BBB permeability [45]. Thus, we investigated the expression of claudin-5 and occludin in different brain areas of symptomatic *Mecp2*^-/y^ mice in comparison with age-matched WT controls. While the expression of claudin-5 was significantly decreased in the cortex and the striatum (Figure 2A,B) of RTT mice, occludin was reduced in the cortex and the cerebellum (Figure 2E,G). No difference in the expression of either proteins was observed in the hippocampus (Figure 2D,H).

**Figure 3:**
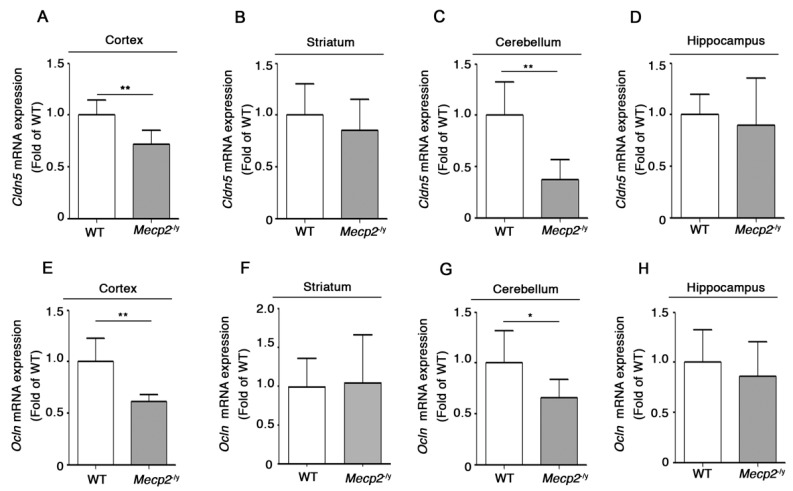
Expression of *Cldn5* and *Ocln* mRNAs is defective in brain tissues of *Mecp2*^-/y^ mice. qPCR analysis of *Cldn5* and *Ocln* in cortex (**A**,**E**), striatum (**B**,**F**), cerebellum (**C**,**G**), and hippocampus (**D**,**H**) of *Mecp2*^-/y^ mice and age-matched WT littermates. Data were normalized to *Gapdh.* Values are represented as mean ± SD. N = 6 for each group of mice. *, *p* < 0.05; **, *p* < 0.01 (two-tailed unpaired *t*-test).

Next, in order to establish whether the aberrant claudin-5 and occludin protein expression was paralleled by any defect in gene expression, we investigated mRNA levels of both *Claudin-5* (*Cldn5*) and *Occludin* (*Ocln*) genes in different brain regions of fully manifest *Mecp2*^-/y^ in comparison with age-matched WT mice. The qPCR analysis showed a significant decrease in *Cldn5* mRNA in the cortex and the cerebellum (Figure 3A,C) and a significantly decreased *Ocln* transcript in the same brain areas of *Mecp2*-null mice compared with the WT counterpart (Figure 3E,G). No differences in the expression of *Cldn5* and *Ocln* were observed in either the striatum or the hippocampus of the same animals (Figure 3B,D,F,H).

### 3.3. Expression of Additional Genes Involved in BBB Structure and Function Is Aberrant in Symptomatic Mecp2^-/y^ Mice

To further clarify the molecular mechanisms potentially underling the increased BBB permeability in RTT mice, we analyzed the expression of a number of additional genes encoding factors implicated in the regulation of BBB structure and function, such as *Claudin-3* (*Cldn3*), *Claudin-12* (*Cldn12*), *Junctional Adhesion Molecule 2* (*Jam2*), *Acquaporin 4* (*Aqp4*), and *Multiple PDZ Domain Protein* (*Mpdz*) [30,34,45,46,47,48].

**Figure 4:**
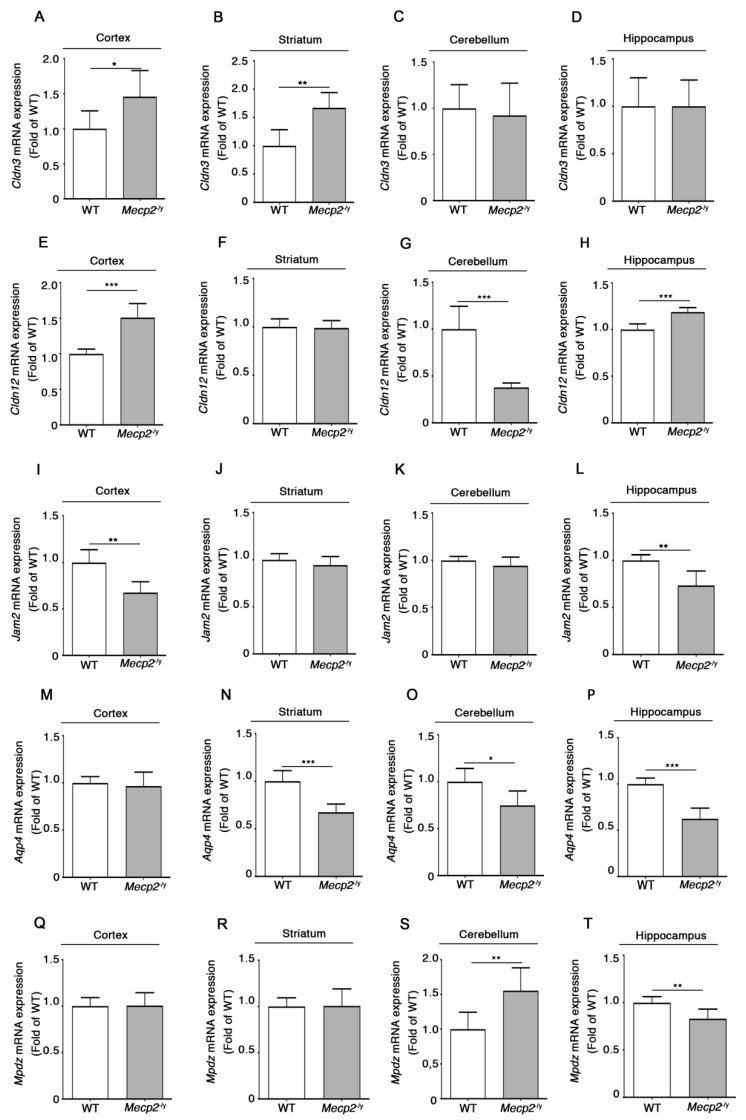
Expression of several genes involved in the regulation of structure and function of the BBB is perturbed in brain tissues of *Mecp2*^-/y^ mice. qPCR analysis of *Cldn3*, *Cldn12, Jam2, Aqp4, and Mpdz* in cortex (**A**,**E**,**I**,**M**,**Q**), striatum (**B**,**F**,**J**,**N**,**R**), cerebellum (**C**,**G**,**K**,**O**,**S**), and hippocampus (**D**,**H**,**L**,**P**,**T**) of *Mecp2*^-/y^ mice and age-matched WT littermates. Data were normalized to *Gapdh.* Values are represented as mean ± SD. N = 6 for each group of mice. *, *p* < 0.05; **, *p* < 0.01, ***, *p* < 0.001 (two-tailed unpaired *t*-test).

The qPCR analysis revealed that each gene displayed a characteristic expression profile depending on the brain region analyzed. The expression of *Cldn3* was significantly higher in the cortex and the striatum of symptomatic *Mecp2*^-/y^ in comparison with WT mice (Figure 4A,B), whereas its levels were unchanged in the cerebellum and the hippocampus (Figure 4C,D).

*Cldn12* mRNA was increased in the cortex and the hippocampus (Figure 4E,H) and strongly reduced in the cerebellum (Figure 4G) of symptomatic *Mecp2*-null mice, whereas no *Cldn12* deregulation was noticed in the striatum of RTT mice (Figure 4F).

A reduced expression of *Jam2* was observed in the RTT cortex and hippocampus (Figure 4I,L), while its mRNA levels were unaffected in the striatum and the cerebellum of *Mecp2*^-/y^ mice (Figure 4J,K).

Moreover, in the absence of *Mecp2*, a decreased expression of *Aqp4* transcripts was detected in the striatal, cerebellar, and hippocampal regions of symptomatic *Mecp2*-null mice (Figure 4N–P), whereas no alterations of *Aqp4* levels were found in the cortex of RTT mice (Figure 4M).

Finally, we observed an opposite expression profile of *Mpdz* mRNA between the cerebellum and the hippocampus of fully manifest *Mecp2*^-/y^ mice (Figure 4S,T), while no *Mpdz* expression changes were detected in the cortex or the striatum of the same animals (Figure 4Q,R).

*Matrix Metallopeptidase 9* (*Mmp9*) is a metalloproteinase known to impact BBB permeability by inducing the degradation of basal lamina and TJs, as well as to stimulate the release of cytokines and free radicals, thus contributing to enhance neuroinflammation [49,50], previously advocated as a possible mechanism for developmental cognitive impairment also in RTT syndrome [51,52]. In order to understand whether *Mmp9* may play a role in the BBB perturbation in RTT mice, the expression of its transcript in symptomatic *Mecp2*^-/y^ mice was assessed.

**Figure 5:**
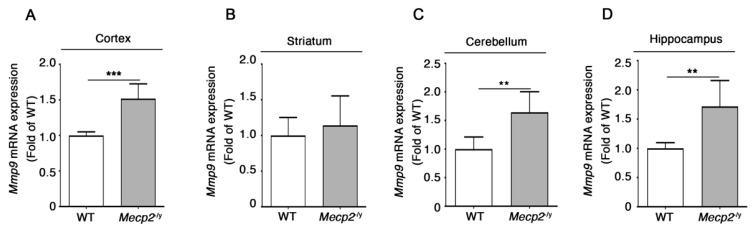
The expression of *Mmp9* is up-regulated in the brain of *Mecp2*^-/y^ mice. qPCR analysis of *Mmp9* in cortex (**A**), striatum (**B**), cerebellum (**C**), and hippocampus (**D**) of *Mecp2*^-/y^ and age-matched WT mice. Data were normalized to *Gapdh.* Values are represented as mean ± SD. N = 6 for each group of mice. **, *p* < 0.01, ***, *p* < 0.001 (two-tailed unpaired *t*-test).

*Mmp9* mRNA levels were increased in the cortex, the cerebellum, and the hippocampus of symptomatic *Mecp2*^-/y^ mice with respect to the WT controls (Figure 5A,C,D), whereas no differences in *Mmp9* transcript levels were detected in the striatum of the same mice (Figure 5B).

## 4. Discussion

The disruption of BBB has been associated with a wide variety of neuropathological conditions, spanning from neurodegenerative diseases [25,26,27,28,29,30,31], neuropsychiatric disorders [34,35], and ASD [32,33]; however, no evidence has been reported specifically for Rett syndrome.

**Figure 6:**
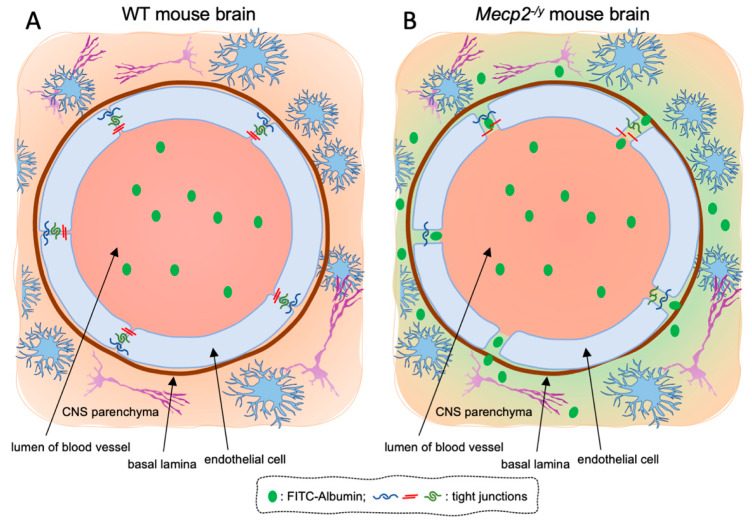
Schematic representation of the blood–brain barrier in WT and *Mecp2*^-/y^ mice. (**A**) In WT brain, the tracer FITC-albumin is retained within the lumen of blood vessels due to the integrity of the TJs between the endothelial cells in the brain parenchyma. (**B**) In *Mecp2*^-/y^ brain, an increased permeability of the BBB, visible as a diffusion of FITC-albumin along perivascular spaces and brain parenchyma, occurs (likely due to disruption of the TJs).

In this study, we provide the first evidence of perturbed BBB integrity in one of the most-used *Mecp2*^-/y^ RTT mouse models. The extravasation of FITC-albumin, observed in the brain parenchyma of *Mecp2*^-/y^ mice, is indicative of the BBB breakdown in RTT syndrome (Figure 6), which ultimately results in increased vascular permeability.

From a molecular standpoint, this “functional” defect was associated with a perturbed expression of the TJ proteins, claudin-5 and occludin, whose expression is critical for BBB functional integrity [53,54]. However, while reduction in the expression of occludin was consistent with decreased gene expression, no correlation between mRNA and protein expression levels for claudin-5 was observed. These findings suggest the existence of multiple levels of regulation of TJ integrity, which may potentially include post-transcriptional mechanisms as well as protein stability and turnover.

Our findings highlighted an aberrant expression of a number of additional genes encoding factors known to be involved in the regulation of BBB stability and functions in RTT mice. Interestingly, depending on the brain region analyzed, we found either down- or up-regulation of specific genes. In particular, *Cldn12*, previously reported to be deregulated in the postmortem cortex and cerebellum from ASD and schizophrenia patients [32], and *Mpdz*, encoding a scaffold protein that regulates TJs, showed a variable gene expression profile in a brain region depending manner, suggesting a specific vulnerability of different brain districts. Considering each single brain region, the unexpected up-regulation of some BBB-related genes, such as *Cldn3*, *Cldn12,* and *Mpdz,* may represent a compensatory response to eventually counteract the down-regulation of the other factors crucial for BBB sealing. Of note, the up-regulation of *Cldn3* and *Cldn12* transcripts has been previously reported in the cortex of ASD subjects [32].

Among the BBB-associated genes found to be deregulated in *Mecp2*^-/y^ mouse brain, we found *Jam2* and *Mpdz*. The *Jam2* gene, which appeared down-regulated in the cortex and the hippocampus of *Mecp2*^-/y^ mice, encodes a structural component of TJs and is involved in the assembling of TJ complex components [30]. *Mpdz*, which displayed an opposite deregulation in the cerebellum and the hippocampus of *Mecp2*^-/y^ mice, encodes one of the intracellular accessory TJ molecules that play an integral function by connecting TJs to the actin cytoskeleton and by modulating the function of these factors [46,55]. Although the physio-pathological role of JAM proteins and MPDZ in the BBB breakdown in neurological diseases is not clear yet, we may suppose that the altered expression of these two factors might contribute to the observed BBB disruption in *Mecp2*-null models.

AQP4 is a water channel localized in the endfeet of astrocytes in contact with blood vessels [56], known to contribute to the maturation of the BBB and the maintenance of its integrity [48,57,58,59]. A recent work illustrates that the restoration of the *Aqp4* expression in a murine model of intracerebral hemorrhage retrieves BBB integrity and inhibits BBB leakage [47]. In line with this evidence, we may hypothesize that decreased *Aqp4* expression observed in the striatum, cerebellum, and hippocampus of *Mecp2*^-/y^ mice might have a role in the BBB breakdown.

Another interesting point of our study is the evidence that the expression of *Mmp9* is increased in RTT brain tissues. A number of studies demonstrate a critical role of matrix metalloproteinases in the pathogenesis of BBB damage [49,60,61,62], possibly through the degradation of basal lamina that surrounds microvessels and the cleavage of occludin [60,63]. However, MMP9 is known to be also involved in the inflammatory response through the activation of cytokines and chemokines [49]. Moreover, the expression of MMP9 is elevated in several neurological conditions, including ischemia, neurodegenerative diseases (e.g., Parkinson’s, Huntington’s, and Alzheimer’s diseases), ASDs, Fragile X syndrome [32,49,64,65], and in neuroinflammatory disorders such as multiple sclerosis, for which MMP9 has been proposed to have a pathogenetic role in the disruption of the BBB [49]. This scenario allows to hypothesize that the enhanced expression of *Mmp9* in the cortex, cerebellum and hippocampus of *Mecp2*-null mice might be indicative of a neuroinflammatory state caused by a BBB breakdown, in line with the evidence of neuroinflammation reported in RTT [51,52]. Studies performed in superoxide dismutase (SOD)1-null mice suggested that oxidative stress might play a role in the alteration of BBB permeability, possibly through the stimulation of MMP9 [65]. This is an interesting point, considering that systemic oxidative stress has been reported in RTT patients [66,67] and oxidative brain damage has been characterized in different RTT mouse models [68]. In this light, and according to the observed augmented expression of *Mmp9*, it is conceivable that increased oxidative stress in the RTT brain might induce the over-production of MMP9 that, in turn, might concur to disrupt BBB.

BBB integrity is required not only to physically block the entrance of pathogens and/or toxic/harmful molecules from circulating blood to the brain parenchyma but it is crucial to also maintain the homeostasis of several metabolites. Thus, it is plausible that any perturbations of BBB might have a deleterious impact on brain metabolism. Interestingly, an untargeted metabolomic analysis in the cortices of *Mecp2*^-/y^ and WT mice revealed an altered content of different metabolites in Rett syndrome [69]. In particular, several amino acids, including glutamate, appeared to be over-represented in the *Mecp2*^-/y^ cortex [69]. Of note, previous in vitro studies suggested that microglia from *Mecp2*^-/y^ mice are responsible for the over-production of glutamate [70], which exerts a neurotoxic effect on dendrites and synapses [70,71]. We might speculate that the disruption of BBB in *Mecp2*^-/y^ mice could be responsible for abnormal metabolism in the brain and impaired excretion of metabolites that are neurotoxic at high levels, such as glutamate, with the consequent exacerbation of the brain damage.

Additional studies are needed to assess any precocious sign of BBB disruption in *Mecp2*^-/y^ mice. This may contribute to the discovery of new potential disease biomarkers and to the identification of novel therapeutic targets for RTT.

## 5. Conclusions

Despite decades of research, the molecular mechanisms by which *MECP2* mutations so deeply affect the normal brain functioning in RTT patients remain still poorly understood. The present study highlighted, for the first time, the alteration of the BBB permeability in the brain of *Mecp2*^-/y^ mice, which might be caused by the deregulated expression of key factors involved in the BBB structure and function. Although our results do not allow to clarify whether perturbed BBB permeability participates in RTT pathogenesis or whether it is a secondary effect of the disease, to our perspective, this study revealed a new molecular hallmark of the disease that may represent a target for yet unexplored therapeutic strategies. However, further studies are needed to fully characterize BBB disruption in RTT and to clarify the pathogenetic role of MeCP2 dysfunction in this scenario.

## Figures and Tables

**Table 1:** List of primers used in this study.

Primer Name	Primer Forward Sequence (5′–3′)	Primer Reverse Sequence (5′–3′)
*Cldn5*	CAGTTAAGGCACGGGTAGCA	GGCACCGTCGGATCATAGAA
*Occludin*	AGACCTGATGAATTCAAACCCAAT	ATGCATCTCTCCGCCATACAT
*Cldn3*	TACAAGACGAGACGGCCAAG	GGGCACCAACGGGTTATAGA
*Jam2*	GGCAAATGGGTTTTCTGCATC	TGGAGAGCCTGTTGGTAGTAGA
*Mpdz*	GTCCTTTTAGTTGGTGTTTGGCA	CGCGTTCTTTCAGCTTGCTT
*Aqp4*	ATCCTCTACCTGGTCACACCT	ATAGTGAACACCAACTGGAAAGTG
*Cldn12*	ACGGCCTTCAATTCTTCCGT	AGACCGGCTCAAACTTCCTG
*Mmp9*	GACGACATAGACGGCATCCAG	GGATAGGCCGTGGGAGGTAT
*Gapdh*	CCAGGAGCGAGACCCCACTA	GGGCGGAGATGATGACCCTT

## Data Availability

The data are available upon reasonable request.
